# Supplementary material for: Anti-Proliferative Potential of Quercetin Loaded Polymeric Mixed Micelles on Rat C6 and Human U87MG Glioma Cells
Source: Pharmaceutics. 2022 Aug 6;14(8):1643. doi: 10.3390/pharmaceutics14081643 (PMC9412540; doi:10.3390/pharmaceutics14081643)
Supplement: Supplementary file 1 [file pharmaceutics-14-01643-s001.zip › pharmaceutics-1773506-supplementary.pdf]

Supplementary File

Anti-Proliferative Potential of Quercetin Loaded Polymeric Mixed Micelles on Rat C6 and Human U87MG Glioma Cells

Sathishbabu Paranthaman [1], Chinnappa A. Uthaiah [2], Riyaz Ali M. Osmani [1], Umme Hani [3], Mohammed Ghazwani [3,4], Ali H. Alamri [3], Adel Al Fatease [3], SubbaRao V. Madhunapantula [2] and Devegowda Vishkante Gowda [1,*]

[1] Department of Pharmaceutics, JSS College of Pharmacy, JSS Academy of Higher Education and Research, Mysuru 570015, Karnataka, India
[2] Centre of Excellence in Molecular Biology and Regenerative Medicine (CEMR) laboratory, (a DST-FIST Sponsored Centre) Department of Biochemistry (a DST-FIST Sponsored Department), JSS Medical College, JSS Academy of Higher Education & Research (JSS AHER), Mysuru - 570015, Karnataka, India
[3] Department of Pharmaceutics, College of Pharmacy, King Khalid University, Guraiger, Abha 61421, Saudi Arabia
[4] Cancer Research Unit, King Khalid University, Guraiger, Abha 61421, Saudi Arabia
[*] Correspondence: dvgowda@jssuni.edu.in

Supplementary Equations

$$Particle\ Size = +716.09520 + 499.87603 * Soluplus - 1038.48627E - TPGS - 4469.14112 * Poloxamer407 + 247.80037 * Soluplus * E - TPGS - 590.50167 * Soluplus * Poloxamer\ 407 + 2403.70370E - TPGS * Poloxamer\ 407 - 222.37313Soluplus^2 + 142.78996E - TPGS^2 + 9815.16333Poloxamer407^2 \ldots\ldots\ldots \text{(S1)}$$

$$PDI = +1.11487 + 0.725355Soluplus - 1.55176E - TPGS - 9.03024Poloxamer\ 407 + 0.369629Soluplus * E - TPGS + 0.241667Soluplus * Poloxamer\ 407 + 1.62407E - TPGS * Poloxamer\ 407 - 0.323134Soluplus^2 + 0.640576E - TPGS^2 + 17.96333 \ldots\ldots\ldots \text{(S2)}$$

$$\%EE = -42.55104 + 117.09552Soluplus + 42.08558E - TPGS - 202.60861\ Poloxamer407 - 45.41852Soluplus * E - TPGS + 172.21667\ Soluplus * Poloxamer\ 407 - 3.55556 * E - TPGS * Poloxamer407 * 35.74400 * Soluplus^2 + 34.08971 * E - TPGS^2 + 36.15000 * Poloxamer\ 407^2 \ldots\ldots\ldots \text{(S3)}$$

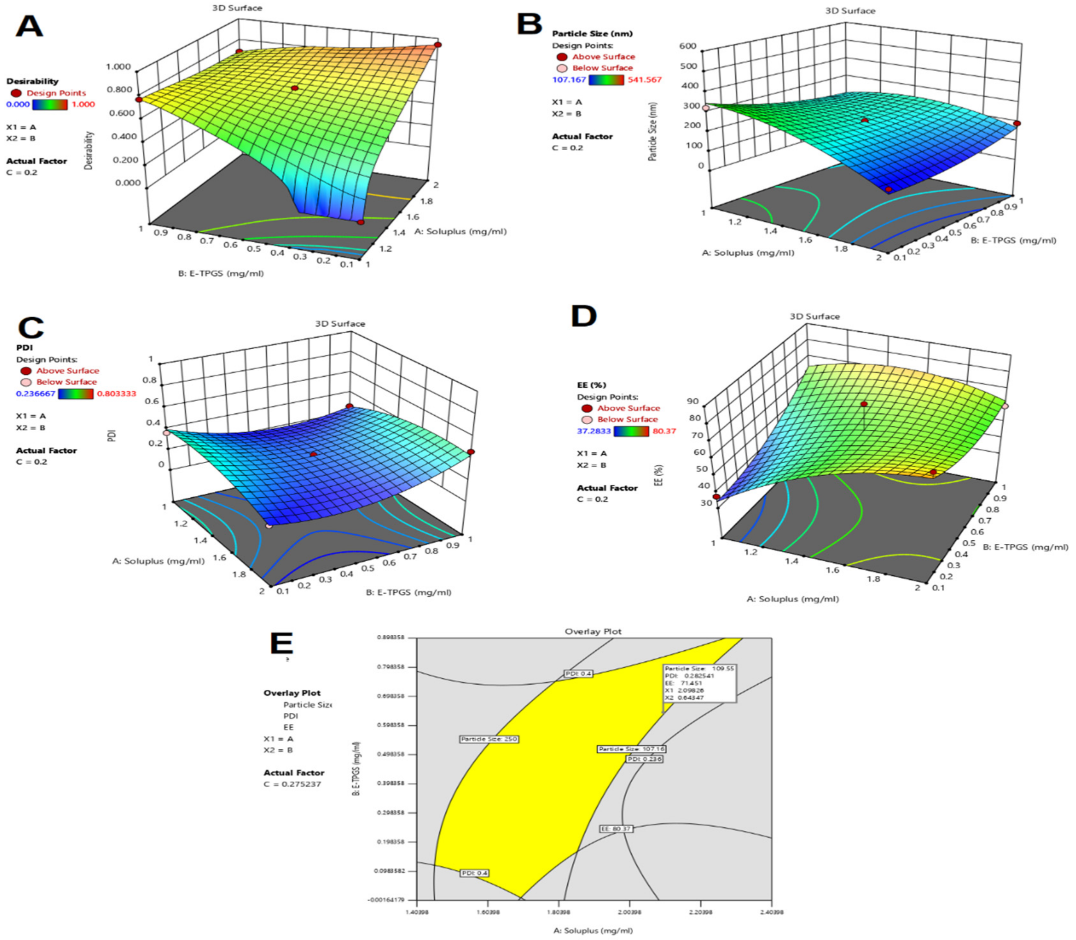

Figure S1: 3D plot analysis of impact of independent variables on dependent variables of prepared Qu-PMMs; A. Desirability, B. Particle size, C. PDI, D. %EE and E. overlay plot of optimized Qu-PMMs.

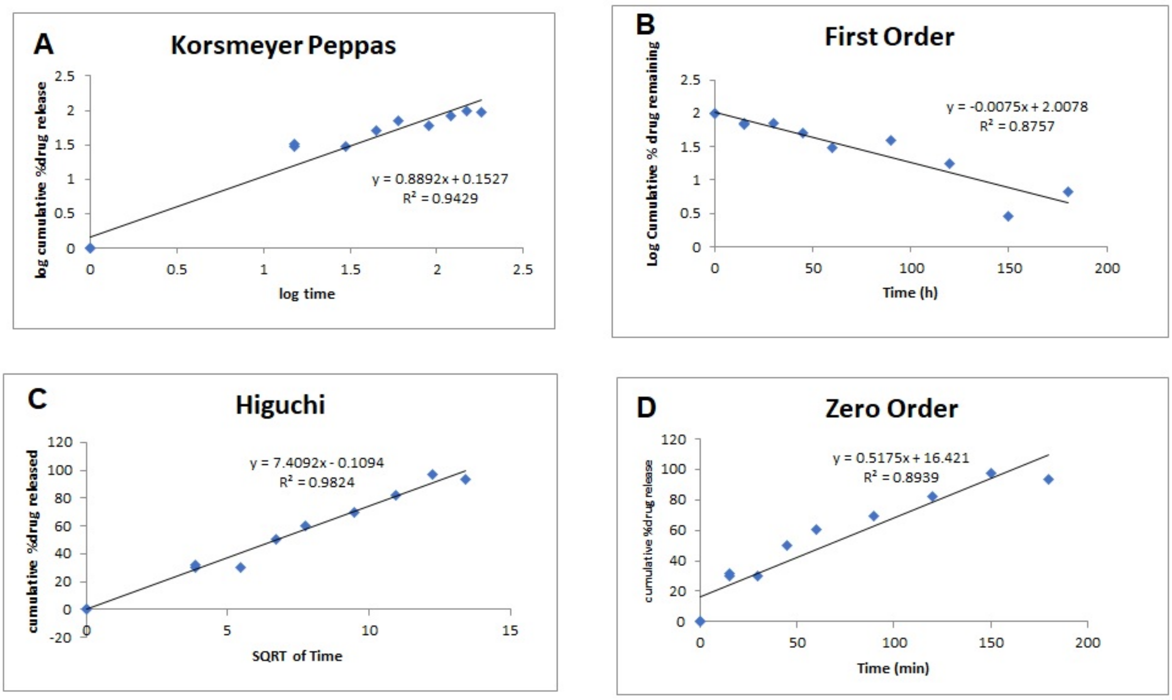

Figure S2. Drug release kinetic model; A. Korsmeyer peppas drug release model; B. First order drug release model; C. Higuchi drug release model; D. Zero order drug release model.
